# Use of anastrozole for breast cancer prevention (IBIS-II): long-term results of a randomised controlled trial

**DOI:** 10.1016/S0140-6736(19)32955-1

**Published:** 2020-01-11

**Authors:** Jack Cuzick, Ivana Sestak, John F Forbes, Mitch Dowsett, Simon Cawthorn, Robert E Mansel, Sibylle Loibl, Bernardo Bonanni, D Gareth Evans, Anthony Howell, IBIS-II investigators

**Affiliations:** a Centre for Cancer Prevention, Wolfson Institute of Preventive Medicine, Queen Mary University London, London, UK; b Australia New Zealand Breast Cancer Trials Group Newcastle, University of Newcastle, Calvary Mater Hospital, Waratah, NSW, Australia; c Ralph Lauren Centre for Breast Cancer Research, Royal Marsden, London, UK; d Breast Care Centre, Southmead Hospital, Bristol, UK; e University Department of Surgery, University of Wales College of Medicine, Cardiff, UK; f German Breast Group, Frankfurt, Germany; g Division of Chemoprevention and Genetics, European Institute of Oncology, Milan, Italy; h Prevent Breast Cancer Unit, Nightingale Breast Screening Centre, Manchester University NHS Foundation Trust, Manchester, UK

## Abstract

**Background:**

Two large clinical trials have shown a reduced rate of breast cancer development in high-risk women in the initial 5 years of follow-up after use of aromatase inhibitors (MAP.3 and International Breast Cancer Intervention Study II [IBIS-II]). Here, we report blinded long-term follow-up results for the IBIS-II trial, which compared anastrozole with placebo, with the objective of determining the efficacy of anastrozole for preventing breast cancer (both invasive and ductal carcinoma in situ) in the post-treatment period.

**Methods:**

IBIS-II is an international, randomised, double-blind, placebo-controlled trial. Postmenopausal women at increased risk of developing breast cancer were recruited and were randomly assigned (1:1) to either anastrozole (1 mg per day, oral) or matching placebo daily for 5 years. After treatment completion, women were followed on a yearly basis to collect data on breast cancer incidence, death, other cancers, and major adverse events (cardiovascular events and fractures). The primary outcome was all breast cancer.

**Findings:**

3864 women were recruited between Feb 2, 2003, and Jan 31, 2012. 1920 women were randomly assigned to 5 years anastrozole and 1944 to placebo. After a median follow-up of 131 months (IQR 105–156), a 49% reduction in breast cancer was observed for anastrozole (85 *vs* 165 cases, hazard ratio [HR] 0·51, 95% CI 0·39–0·66, p<0·0001). The reduction was larger in the first 5 years (35 *vs* 89, 0·39, 0·27–0·58, p<0·0001), but still significant after 5 years (50 *vs* 76 new cases, 0·64, 0·45–0·91, p=0·014), and not significantly different from the first 5 years (p=0·087). Invasive oestrogen receptor-positive breast cancer was reduced by 54% (HR 0·46, 95% CI 0·33–0·65, p<0·0001), with a continued significant effect in the period after treatment. A 59% reduction in ductal carcinoma in situ was observed (0·41, 0·22–0·79, p=0·0081), especially in participants known to be oestrogen receptor-positive (0·22, 0·78–0·65, p<0·0001). No significant difference in deaths was observed overall (69 *vs* 70, HR 0·96, 95% CI 0·69–1·34, p=0·82) or for breast cancer (two anastrozole *vs* three placebo). A significant decrease in non-breast cancers was observed for anastrozole (147 *vs* 200, odds ratio 0·72, 95% CI 0·57–0·91, p=0·0042), owing primarily to non-melanoma skin cancer. No excess of fractures or cardiovascular disease was observed.

**Interpretation:**

This analysis has identified a significant continuing reduction in breast cancer with anastrozole in the post-treatment follow-up period, with no evidence of new late side-effects. Further follow-up is needed to assess the effect on breast cancer mortality.

**Funding:**

Cancer Research UK, the National Health and Medical Research Council Australia, Breast Cancer Research Foundation, Sanofi Aventis, and AstraZeneca.

## Introduction

Early work on therapeutic prevention of breast cancer has focused on selective oestrogen receptor modulators (SERMs), such as tamoxifen and raloxifene, which show anti-oestrogenic effects on the breast, as well as agonistic or antagonistic effects on some other organs. In a meta-analysis of several SERMs,^1^ a 38% reduction in all breast cancer was observed, driven by 50% reduction of oestrogen receptor-positive tumours, but no effect on oestrogen receptor-negative tumours. Long-term follow up of two of these trials has shown that the effects of tamoxifen continue with a constant 29% annual preventive effect for at least 15 years after completion of treatment.^2^, ^3^

A greater short-term reduction in breast cancer incidence was seen in two trials using, the aromatase inhibitors, anastrozole^4^ and exemestane.^5^ The MAP.3 trial^5^ compared exemestane with placebo in postmenopausal women at high risk of developing breast cancer and found a significant reduction in the incidence of all breast cancer by 53% and a 65% reduction in invasive breast cancer after a median follow-up of 35 months. However, all women were unblinded after the initial publication, so it was not possible to study a post-treatment effect, as has been seen with tamoxifen.^2^

The International Breast Cancer Intervention Study II (IBIS-II) was initiated in 2003 and recruited postmenopausal women without breast cancer but at high risk of developing it to receive either anastrozole (1 mg daily) or matching placebo. The first analysis after a median follow-up of 60 months (IQR 36–85) reported a significant reduction in incidence of 53% for all breast cancer (including ductal carcinoma in situ).^4^ A 58% reduction in incidence of invasive oestrogen receptor-positive breast cancer and a 70% reduction in incidence of ductal carcinoma in situ was observed for anastrozole. As reported in adjuvant trials,^6^, ^7^ the main adverse events with anastrozole were fractures, joint-related effects, and menopausal symptoms, which are associated with an almost complete elimination of oestrogen in postmenopausal women using aromatase inhibitors.

**Research in context**

**Evidence before this study**

At the time of the initial publication of this trial, we searched PubMed for reports published in English between Jan 1, 1980, and May 30, 2013. We used the search terms “breast cancer”, “prevention”, and “aromatase inhibitor”. Only one other prevention trial using an aromatase inhibitor had been reported and it has been discussed. However, we identified several adjuvant trials using aromatase inhibitors in which contralateral tumours were reported. We also identified an overview of selective oestrogen receptor modulators for breast cancer prevention. Two large trials in which aromatase inhibitors are being assessed for prevention of ductal carcinoma were also identified. For this Article, we repeated the search up to Oct 30, 2019. A US Preventive Services Task Force review was found, but no new studies on breast cancer prevention with aromatase inhibitors were identified.

**Added value of this study**

We report results of the randomised IBIS-II trial on the extended duration of benefit of anastrozole in preventing breast cancer up to 12 years after entry and indicate for the first time a long-term benefit, which is larger than that seen for tamoxifen in this period. No excess of fractures, other cancers, cardiovascular disease, or death from any specific cause was seen in the extended follow-up. The number needed to treat to prevent one breast cancer has been reduced to 29.

**Implications of all the available evidence**

Our results provide additional support for the use of anastrozole as the treatment of choice for breast cancer risk reduction in most postmenopausal women at high risk of developing breast cancer. Its use has been supported by the National Institute for Health and Care Excellence in the UK and the US Preventive Services Task Force. Identification of women at high risk of early symptoms of oestrogen depletion and their management remains a challenge.

A long-term term reduction of breast cancer incidence for anastrozole or any aromatase inhibitor has not been established, as it has for tamoxifen.^2^, ^3^ Such a result is likely to substantially improve the benefit-risk ratio, as side-effects are uncommon after treatment cessation. The objective of this study was to determine the long-term efficacy of anastrozole for preventing breast cancer (both invasive and ductal carcinoma in situ) in the post-treatment period.

## Methods

### Study design and participants

IBIS-II is an international, randomised, double-blind, placebo-controlled trial. Detailed study design and inclusion and exclusion criteria have previously been reported.^4^ In brief, high-risk postmenopausal women aged 40–70 years were recruited between Feb 2, 2003, and Jan 31, 2012, in 153 breast cancer treatment centres across 18 countries (appendix p 3). Specific risk criteria for entry were broad and have previously been reported.^4^ They were designed to include women aged 45–60 years who had a relative risk of breast cancer that was at least twice as high as that in the general population, those aged 60–70 years who had a risk that was at least 1·5 times higher, and those aged 40–44 years who had a risk that was at least four times higher. The exclusion criteria were being premenopausal, previous breast cancer including ductal carcinoma in situ diagnosed more than 6 months before trial entry, current or previous tamoxifen, raloxifene, or other SERM use for more than 6 months, or participation in IBIS-I, unless off-trial therapy for at least 5 years, intention to continue using oestrogen-based hormone replacement therapy, or previous or planned prophylactic mastectomy.

The trial was approved by the UK North West Multi-Centre Research Ethics Committee and was done in accordance with the Declaration of Helsinki (1996 revision), under the principles of good clinical practice. All participants provided written, informed consent to join the study, provide baseline and follow-up blood samples, and have their past and future health records examined, including access to mammograms and pathology material.

### Randomisation and masking

Consenting eligible women were randomly assigned (1:1) to either anastrozole (1 mg per day, oral) or matching placebo daily for 5 years. Randomisation was stratified by country. All participants and medical personnel were blinded to treatment allocation, which was only held by the central study statistician. Unblinding was only permitted if the participant developed breast cancer, when a clinician considered there to be valid medical or safety reasons, or the participant requested unblinding. Treatment allocation still remains largely blinded for investigators and participating women who have not developed breast or any other cancer (81·3% anastrozole *vs* 76·7% placebo, p=0·0053). A further analysis was planned to take place around 5 years after the last report,^4^ and this analysis is provided 6 years after that report. The decision to analyse the data was made without looking at the results beforehand.

### Procedures

After treatment completion, women were followed on a yearly basis to collect data on breast cancer incidence, death, other cancers, and major adverse events (cardiovascular events, fractures). In the UK, these events were also collected through cancer registries and National Health Services (NHS Digital). In non-UK centres, annual questionnaire or annual clinic visits were used to collect these data.

### Outcomes

The primary outcome was the development of histologically confirmed breast cancer—either invasive or non-invasive (ductal carcinoma in situ). Secondary outcomes were oestrogen receptor-positive breast cancer, breast cancer mortality, other cancers, cardiovascular disease, fractures, and all-cause mortality. Exploratory analyses reported treatment effects by more detailed breast cancer type, specific baseline patient characteristics (age, body-mass index [BMI], previous use of hormone replacement therapy, and previous lobular carcinoma in situ or atypical hyperplasia), and other major cancers by site.

### Statistical analysis

All analyses were done on an intention-to-treat basis, including all randomly assigned patients. Analyses of the efficacy endpoints were based on hazard ratios (HRs) using Cox proportional hazard models,^8^, ^9^ with corresponding 95% CIs, and survival curves were estimated using the Kaplan-Meier method.^10^ Only major adverse effects (other cancers, cardiovascular events, fractures, and deaths) were routinely collected after 5 years in all patients. Side-effects and secondary outcomes were compared between treatment groups using odds ratios (ORs) and Fisher exact significance tests. All p values were two-sided. All analyses were done using STATA version 15.1. This trial is registered as an International Standard Randomised Controlled Trial, number ISRCTN31488319.

### Role of the unding source

The funder of the study had no role in study design, data collection, data analysis, data interpretation, or writing of the report. The corresponding author had full access to all the data in the study and had final responsibility for the decision to submit for publication.

## Results

All women randomly assigned to treatment (N=3864, 1920 anastrozole and 1944 placebo) have been included in this analysis. 3704 (95·9%) were still at risk of developing breast cancer after the 5-year treatment period (1866 anastrozole, 1838 placebo) and follow-up is ongoing (appendix p 3). Median follow-up for this analysis was 131 months (IQR 106–156), and 41 295 women-years of follow-up have been accrued (anastrozole 20 803, placebo 20 491), of which 22 367 women-years were accrued after 5 years of follow-up (anastrozole 11 339, placebo 11 028). Median age at study entry was 59·4 years (IQR 55·0–63·4), 1893 women (47·0%) had used hormone replacement therapy before entering the trial, and 2631 (68·1%) had a BMI of more than 25kg/m^2^. Other baseline demographics are shown in the appendix (p 1).

**Figure 1:**
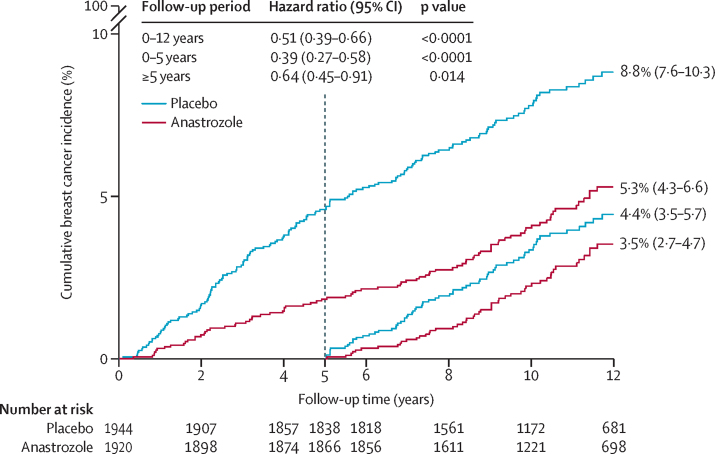
Cumulative incidence for all breast cancer by treatment allocation and follow-up period

250 breast cancers have been reported (85 anastrozole [4·4%] *vs* 165 placebo [8·5%]; table 1), with a highly significant 49% reduction for all breast cancer with anastrozole (HR 0·51, 95% CI 0·39–0·66, p<0·0001). The reduction in incidence in the first 5 years of follow-up was 61% (0·39, 0·27–0·58, p<0·0001), and a smaller but still significant 37% reduction (0·64, 0·45–0·91, p=0·014) was seen in subsequent years, which was still larger than that seen for tamoxifen in previous trials. The effects in the two periods were not significantly different (p=0·087) and a Hosmer-Lemeshow test for non-proportional hazards was not significant (p=0·073). After 12 years of follow-up, the estimated risk of developing breast cancer was 8·8% (IQR 7·6–10·3) in the placebo group compared with 5·3% (4·3–6·6) in the anastrozole group (figure 1), and the number needed to treat for 5 years to prevent one breast cancer was 29.

**Table 1:** Number of breast cancer events and hazard ratios

		**Number of events, anastrozole *vs* placebo**	**Hazard ratio (95% CI)**	**p value**	**p**_heterogeneity_*
Overall		85 *vs* 165	0·51 (0·39–0·66)	<0·0001	..
	0–5 years	35 *vs* 89	0·39 (0·27–0·58)	<0·0001	0·087
	>5 years	50 *vs* 76	0·64 (0·45–0·91)	0·014	..
Invasive oestrogen receptor-positive		48 *vs* 103	0·46 (0·33–0·65)	<0·0001	..
	0–5 years	20 *vs* 51	0·39 (0·23–0·66)	<0·0001	0·43
	>5 years	28 *vs* 52	0·52 (0·33–0·83)	0·0062	..
All ductal carcinoma in situ		13 *vs* 31	0·41 (0·22–0·79)	0·0081	..
	0–5 years	5 *vs* 17	0·29 (0·11–0·80)	0·016	0·43
	>5 years	8 *vs* 14	0·56 (0·23–1·32)	0·18	..

* 0–5 years *vs* >5 years.

**Figure 2:**
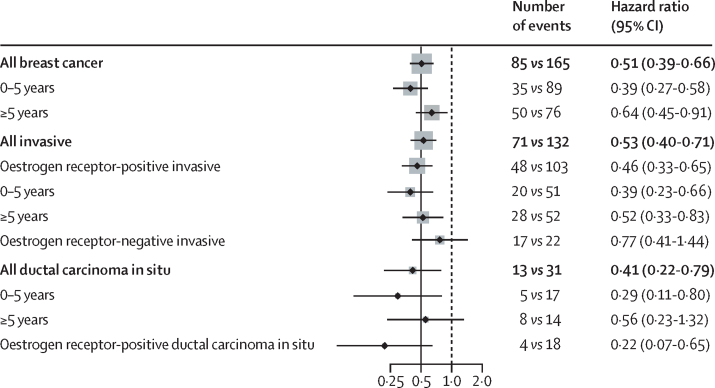
Hazard ratios for subgroup analyses by follow-up period Grey squares indicate the amount of information available for this comparison, largely based on the number of events.

Overall 203 (81·2%) of the breast cancers were invasive, and 151 (74·4%) of these were reported as oestrogen receptor-positive. A 54% reduction in incidence with anastrozole was observed for oestrogen receptor-positive cancers (HR 0·46, 95% CI 0·33–0·65, p<0·0001), with a larger 61% reduction in the first 5 years (0·39, 0·23–0·66, p<0·0001; figure 2), followed by a 48% reduction (0·52, 0·33–0·83, p=0·0062). A small, non-significant reduction in incidence was observed for invasive oestrogen receptor-negative breast cancer in the anastrozole group (0·77, 0·41–1·44, p=0·41; figure 2). A significant reduction in incidence for anastrozole was also found for ductal carcinoma in situ (0·41, 0·22–0·79, p=0·0081), in particular for lesions known to be oestrogen-positive (0·22, 0·07–0·65, p=0·0062; figure 2).

No clear heterogeneity or trend was observed for differences in the preventive effect of anastrozole by other tumour characteristics (appendix p 2). Anastrozole reduced incidence of invasive HER2-negative cancers by 43% (HR 0·57, 95% CI 0·41–0·78), which was similar to that for invasive HER2-positive cancers (0·52, 0·23–1·17; appendix p 2).

Exploratory analyses of baseline characteristics did not show any significant heterogeneity by age, BMI, previous hormone replacement therapy use, or previous lobular carcinoma in situ or atypical hyperplasia (appendix p 2). Reductions in incidence did not differ between treatment groups for women with a BMI of more than 30 kg/m^2^ and or who took hormone replacement therapy before trial entry.

Overall, a 28% reduction in cancer incidence at non-breast sites occurred (147 *vs* 200 cases, OR 0·72, 95% CI 0·57–0·91, p=0·0042; table 2). Secondary analyses showed that this reduction was driven largely by a reduction in the incidence of non-melanoma skin cancer (43 *vs* 73 cases, 0·59, 0·39–0·87, p=0·0058), and no effect on other specific cancers was apparent (table 2). In particular, no reduction in the incidence of endometrial cancer occurred due to oestrogen deprivation from anastrozole, although oestrogen is thought to be a major driver of this cancer.^11^ Additionally, the early reduction seen for colorectal cancers^4^ has not been extended with longer follow-up.

**Table 2:** Cancers other than breast

		**Anastrozole N=1920, n (%)**	**Placebo, N=1944, n (%)**	**Odds ratio (95% CI)**
Total		147 (7·1%)	200 (9·8%)	0·72 (0·57–0·91)
Skin		52 (2·7%)	85 (4·4%)	0·61 (0·42–0·88)
	Non-melanoma	43 (2·2%)	73 (3·8%)	0·59 (0·39–0·87)
	Melanoma	9 (0·5%)	12 (0·6%)	0·76 (0·28–1·97)
Gynaecological		14 (0·7%)	20 (1·0%)	0·71 (0·33–1·47)
	Endometrial	5 (0·3%)	7 (0·4%)	0·72 (0·18–2·65)
	Ovarian	7 (0·4%)	10 (0·5%)	0·71 (0·23–2·06)
Respiratory		13 (0·7%)	13 (0·7%)	1·01 (0·43–2·38)
	Lung	11 (0·6%)	12 (0·6%)	0·93 (0·37–2·30)
Gastrointestinal		24 (1·3%)	33 (1·7%)	0·81 (0·45–1·43)
	Colorectal	11 (0·6%)	16 (0·8%)	0·69 (0·29–1·60)

No effect was seen on any other major adverse event (table 3). In particular, there was no excess of fractures overall (380 *vs* 373, OR 1·04, 95% CI 0·88–1·22). A small non-significant increase in number of events during the active treatment period (198 *vs* 186, 1·09, 0·87–1·35) was counterbalanced by slight reduction of events after treatment was completed (182 *vs* 187, 0·98, 0·79–1·23). Myocardial infarctions were evenly distributed between treatment groups (table 3), and no differences in number of events were observed in the first 5 years (eight *vs* eight) or in follow-up after treatment (eight *vs* six). Numbers of deep vein thromboses were slightly increased in the placebo group, with no differences observed in the two periods (table 3). Cases of pulmonary embolism were non-significantly more frequent with anastrozole but no differences were observed during treatment compared with after treatment (table 3). Transient ischaemic attacks and strokes were non-significantly more common with anastrozole compared with placebo (46 *vs* 36, p=0·24).

**Table 3:** Major adverse events

	**Anastrozole, N=1920, all years (>5 years)**	**Placebo, N=1944, all years (>5 years)**	**Odds ratio (95% CI), all years**
Fractures	380 (182)	373 (186)	1·04 (0·88–1·22)
Myocardial infarction	16 (8)	14 (8)	..
Deep vein thrombosis*	13 (6)	17 (5)	..
Pulmonary embolism	17 (11)	12 (7)	..
Transient ischaemic attack†	24 (14)	20 (9)	..
Stroke	23 (15)	17 (9)	..

* In the absence of pulmonary embolism.

† In the absence of stroke. Numbers in parentheses refer to events occurring in the post-treatment period (>5 year follow-up).

Other less serious side-effects observed in the first 5 years during treatment with anastrozole,^4^ including arthralgia, joint stiffness, hot flushes, night sweats, vulvovaginal dryness, hypertension, and dry eyes, were not collected after the 5-year treatment period. However, even within the treatment period they were most common in the first year of treatment, so it is unlikely that there will be material differences in the post-treatment period. All participants have now completed treatment and full 5-year adherence was 74·6% for anastrozole compared with 77·0% for placebo (HR 0·89, 95% CI 0·79–1·01, p=0·081; appendix p 4), indicating that side-effects of anastrozole had little effect on treatment adherence.

139 (3·6%) women died during the study (69 anastrozole *vs* 70 placebo; table 4), with no difference between the two treatment groups (HR 0·96, 95% CI 0·69–1·34, p=0·82). Overall, no effect of anastrozole was seen for breast cancer-specific mortality (three anastrozole *vs* two placebo), but numbers are very small. Given the small number of deaths and the relatively young median age at entry (59·4 years), substantially longer follow-up will be needed to determine whether anastrozole affects breast cancer and other cause mortality. Deaths from cancers other than breast did not differ between treatment groups (p=0·39).

**Table 4:** Specific causes of death

	**Anastrozole, N=1920, n (%)**	**Placebo, N=1944, n (%)**	**Hazard ratio (95% CI)**
All	69 (3·6%)	70 (3·6%)	0·96 (0·69–1·34)
Breast cancer	2 (0·1%)	3 (0·2%)	0·64 (0·11–3·88)
Other cancer	27 (1·4%)	34 (1·8%)	0·77 (0·47–1·28)
Cardiovascular	13 (0·7%)	9 (0·5%)	1·41 (0·60–3·31)
Other or unknown	27 (1·4%)	24 (1·2%)	1·10 (0·63–1·91)

## Discussion

This updated analysis of the IBIS-II trial provides additional support for the use of anastrozole in breast cancer prevention for high-risk postmenopausal women. The large 61% reduction in breast cancer incidence in the first 5 years has been maintained in subsequent follow-up to 12 years. The significant 36% reduction during post-treatment follow-up was not significantly smaller than during treatment, and still greater than that observed for tamoxifen, which has produced a roughly constant 29% reduction for 20 years.^2^ The number needed to treat to prevent one breast cancer during the first 12 years of follow-up was 29, which compares favourably with the 58 needed for tamoxifen at that time.^2^ Very few deaths from breast cancer have occurred to date, but it is too early to expect an effect on this outcome, which is a limitation of this analysis. The reduction with anastrozole was primarily seen in oestrogen receptor-positive cancers, which suggests that the effect on mortality will be smaller than that for incidence. The effects were greatest for oestrogen receptor-positive tumours, but an unexpected and non-significant 27% reduction was also seen for receptor-negative cancers, which will need further follow-up to validate.

The previously observed^4^ reduction of other cancers with anastrozole, notably non-melanoma skin cancer, has continued with longer follow up. No other side-effects have been identified with longer follow-up, and the small 11% excess of fractures during the active treatment period has not continued after 5 years of follow-up. A limitation of this analysis is that routine collection of less serious side-effects was not done after the 5-year treatment period.

All women have completed the active follow-up period of the trial and now are followed for long-term outcomes by various methods. In the UK, long-term data are collected through national registries for deaths, cancers, and major predefined adverse events, so we are confident that data are complete. Additionally, we still collect data through annual questionnaires where appropriate. For international centres, annual questionnaires were used to collect information on all primary and secondary outcomes. However, data on lesser side-effects, such as hot flushes and musculoskeletal events, were only collected during the 5-year treatment period.

In conclusion, these updated results show a continuing long-term effect of 5 years of anastrozole treatment in preventing breast cancer in high-risk postmenopausal women. No new major adverse events were identified. Overall, our data substantially strengthen the findings from our initial report after 5 years of follow-up.^4^ In the UK, the National Institute for Health and Care Excellence has now recommended the use of anastrozole for breast cancer prevention in high-risk postmenopausal women,^12^ and in the USA, the US Preventive Services Task Force has also supported its use.^13^ The benefits of anastrozole, in terms of the reduction in risk of breast cancer in high-risk postmenopausal women, extend beyond the 5-year treatment period.

## Data sharing

Data will be available according to IBIS-II's data sharing plan. Requests for specific analyses or data can be submitted by email to j.cuzick@qmul.ac.uk. Details for data sharing policy and application process can be found on the website of Queen Mary University London.
