
JOURNAL INFORMATION
==============================
NLM Title Abbreviation: Lancet
Journal ID: Lancet
NLM Journal ID: 2985213R

Lancet (London, England)

ISSN: 0140-6736
EISSN: 1474-547X

ARTICLE INFORMATION
==============================
PMCID: PMC7273897
PMID: 32061297
DOI: 10.1016/S0140-6736(20)30323-8
Article ID: S0140-6736(20)30323-8
Article version: 1
Subjects: Article

Department of Error

Print publication date: 2020 Feb 15
Volume: 395
Issue: 10223
First page: 496
Last page: 496
Copyright: © 2020 The Author(s). Published by Elsevier Ltd.
Copyright year: 2020
Copyright holder: Elsevier Ltd
License: This is an open access article under the CC BY license (http://creativecommons.org/licenses/by/4.0/).
License URL: https://creativecommons.org/licenses/by/4.0/

Erratum for: Use of anastrozole for breast cancer prevention (IBIS-II): long-term results of a randomised controlled trial 395 10218 11 1 2020 117 122 Lancet (London, England) 10.1016/S0140-6736(19)32955-1 PMC6961114 31839281

==============================
Cuzick J, Sestak I, Forbes JF, et al. Use of anastrozole for breast cancer prevention (IBIS-II): long-term results of a randomised controlled trial. Lancet 2020; 395: 117–22—The Acknowledgments of this Article have been updated as of Feb 13, 2020.

