# Department of Error

**DOI:** 10.1016/S0140-6736(21)00427-X

**Published:** 2021-02-27

*Cuzick J, Sestak I, Forbes JF, et al. Use of anastrozole for breast cancer prevention (IBIS-II): long-term results of a randomised controlled trial.* Lancet *2020; **395:** 117–22*—In this Article, Bernardo Bonanni's affiliation should have been “Division of Cancer Prevention and Genetics, IEO, European Institute of Oncology IRCCS, Milan, Italy”. This correction has been made to the online version as of Feb 25, 2021.

