# Supplementary material for: Use of anastrozole for breast cancer prevention (IBIS-II): long-term results of a randomised controlled trial
Source: Lancet. 2020 Jan 11;395(10218):117–22. doi: 10.1016/S0140-6736(19)32955-1 (PMC6961114; doi:10.1016/S0140-6736(19)32955-1)
Supplement: Supplementary appendix [file mmc1.pdf]

THE LANCET

Supplementary appendix

This appendix formed part of the original submission and has been peer reviewed. We post it as supplied by the authors.

Supplement to: Cuzick J, Sestak I, Forbes JF, et al. Use of anastrozole for breast cancer prevention (IBIS-II): long-term results of a randomised controlled trial. *Lancet* 2019; published online Dec 12. http://dx.doi.org/10.1016/S0140-6736(19)32955-1.

Supplementary Table 1: Number of breast cancer events and hazard ratios according to treatment allocation and follow-up period.

	**Anastrozole (N=1920)**	**Placebo (N=1944)**
Age (years), median (IQR)	59·5 (55·0-63·5)	59·4 (55·1-66·3)
Age at menarche (years), median (IQR)	13·0 (12·2-14·0)	13·0 (12·2-14·0)
Age at first child birth (years), median (IQR)	24 (21-27)	24 (21-27)
Age at menopause (years), median (IQR)	50 (45-52)	49 (45-52)
BMI, kg/m^2	27·4 (24·2-31·1)	27·3 (24·4-31·2)
Previous HRT use	893 (47·0%)	910 (47·2%)
Hysterectomy	631 (33·2%)	656 (34·0%)
**Family history (non-exclusive)**		
Two or more relatives with breast/ovarian cancer	956 (49·8%)	938 (48·3%)
One relative with breast cancer at age ≤ 50	675 (35·3%)	653 (33·7%)
One relative with bilateral breast cancer	166 (8·6%)	141 (7·3%)
LCIS/Atypical hyperplasia	154 (8·0%)	190 (9·8%)
DCIS (treated with mastectomy)	160 (8·3%)	166 (8·5%)

IQR=Interquartile Range, kg=kilogram, m=meter, LCIS=lobular carcinoma in situ, DCIS=ductal carcinoma in situ

Supplementary Table 2: Number of invasive breast cancer events and hazard ratios according to treatment allocation and subgroups.

	**Number of events (anastrozole vs. placebo)**	**HR (95% CI)**	**P-heterogeneity/P-trend (1df)**
**Nodal status**			
Negative	35 vs. 88	0·39 (0·27-0·58)	
Positive	26 vs. 32	0·80 (0·48-1·34)	0·083
**Tumour grade**			
Low	14 vs. 17	0·81 (0·40-1·65)	
Intermediate	33 vs. 72	0·45 (0·30-0·68)	
High	21 vs. 39	0·53 (0·31-0·91)	0·18
Tumour size			
≤ 10mm	17 vs. 41	0·41 (0·23-0·72)	
10-20mm	26 vs. 48	0·53 (0·33-0·86)	
> 20mm	28 vs. 43	0·64 (0·40-1·03)	0·31
HER2 status			
Negative	58 vs. 101	0·57 (0·41-0·78)	
Positive	9 vs. 17	0·52 (0·23-1·17)	0·86
Age			
≤ 55 years	17 vs. 32	0·51 (0·28-0·91)	
55-60 years	13 vs. 44	0·32 (0·17-0·59)	
> 60 years	41 vs. 56	0·70 (0·47-1·05)	0·5
BMI			
≤ 25 kg/m^2	17 vs. 28	0·59 (0·32-1·07)	
25-30 kg/m^2	27 vs. 45	0·61 (0·38-0·98)	
>30 kg/m^2	27 vs. 57	0·46 (0·29-0·73)	0·66
HRT			
Never	36 vs. 74	0·48 (0·32-0·71)	
Prior	35 vs. 58	0·60 (0·39-0·91)	0·46
Benign breast disease			
LCIS or AH	8 vs. 23	0·34 (0·15-0·77)	
No LCIS or AH	63 vs. 109	0·57 (0·42-0·78)	0·21

HR=Hazard Ratio, CI=Confidence Intervals, df=degrees of freedom, BMI=Body Mass Index, LCIS=Lobular Carcinoma in Situ, AH=Atypical Hyperplasia, HRT=Hormonal Replacement Therapy

Supplementary Figure 1: CONSORT diagram

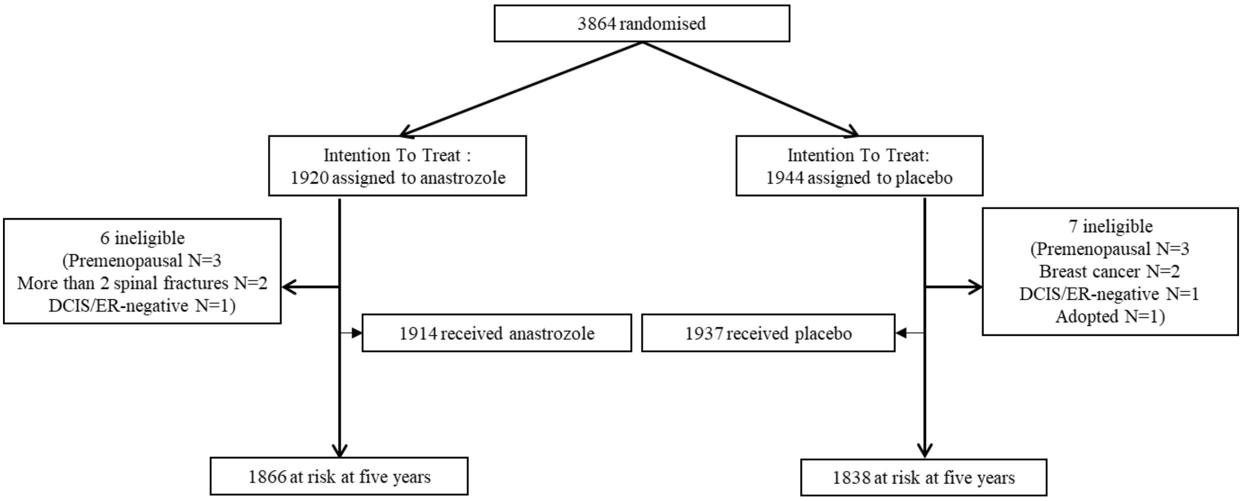

Supplementary Figure 2: Five-year adherence (%) according to treatment allocation.

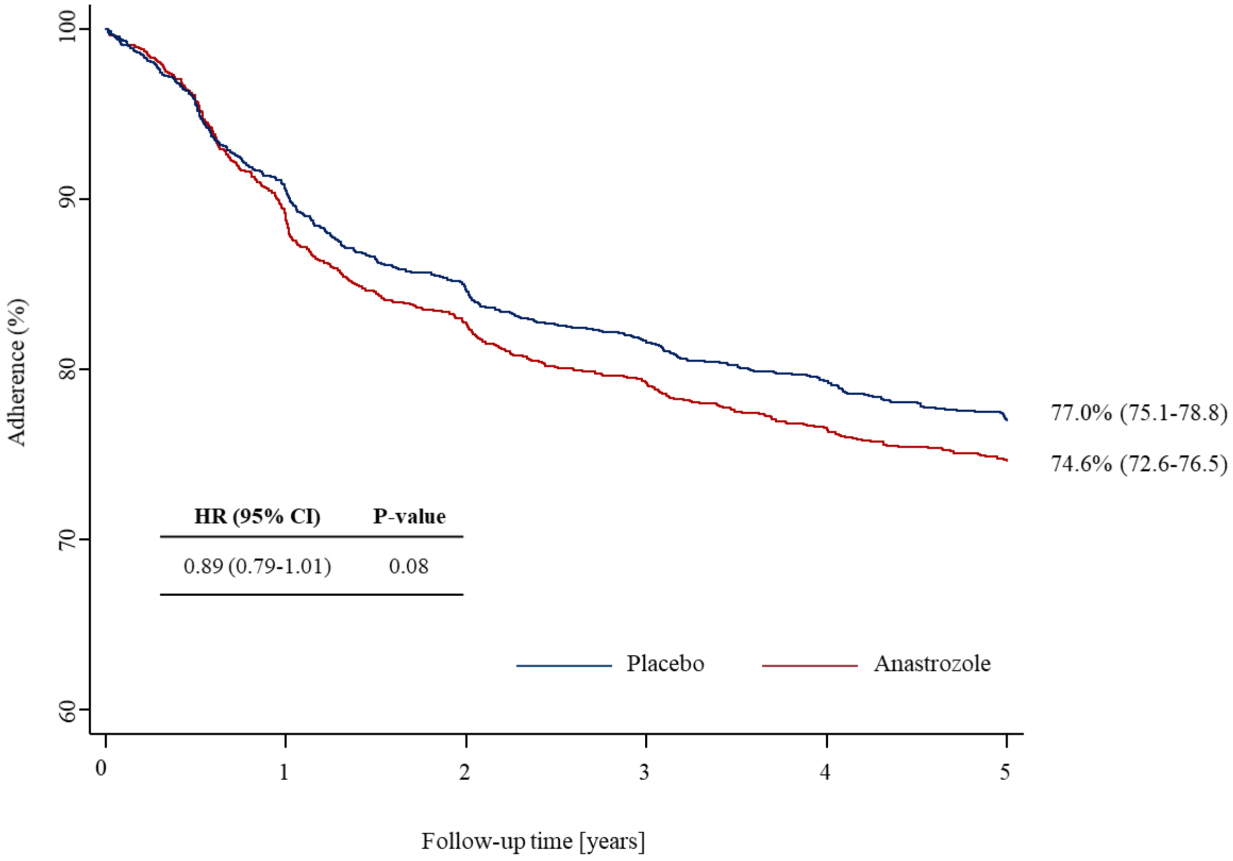

Appendix

Independent Trial Steering Committee:

Richard Sainsbury – Chairman (IOW NHS Trust; London, United Kingdom)
Judy Garber (Dana Farber Cancer Institute, Boston, United States)
Nick Zdenkowski (University of Newcastle, Newcastle, Australia)
Rhian Gabe (Centre for Cancer Prevention, Queen Mary University, London, United Kingdom)

Principal Investigators and Local Coordinators:

Vesna Bjelic-Radisic, Dept. of Obstet. and Gynecology, Graz, Austria
Michael Fridrik, General Hospital Linz, Linz, Austria
Manuela Gili, Austria National Coordinating Centre, Austria
Richard Greil, Third Medical Dept., Salzburg, Austria
Verena Göschl, Austria National Coordinating Centre, Austria
Dietmar Heck, Ordensklinikum Linz Barmherzige Schwestern, KH BHS Linz - Department Chirurgische Abteilung , Austria
Wilfried Horvath, Dept. of Surgery, Gussing, Austria
Raimund Jakesz, Department of Surgery, Vienna University Medical School, Vienna, Austria
Petra Luft, Salzburg Cancer Research Institute (SCRI) Center for Clinical Cancer and Immunology Trials (CCCIT), Salzburg, Austria
Angela Ramoni, Dept. of Obst. and Gyn, Innsbruck, Austria
Arno Reichenauer, Dept. of Surgery, Sankt Veit, Austria
Christian Singer, Medical University of Vienna, Department Allgemeine Gynäkologie, Medical University of Vienna, Austria
Katharina Steiner, Austrian Breast and Colorectal Cancer Study Group, Austria National Coordinating Centre, Austria
Michael Stierer, Univ. Prof. Dr. Michael Stierer, Austria National Coordinating Centre, Austria
Josef Thaler, Hospital Klinikum Kreuzschwestern Wles, Oberösterreich, Austria
Thomas Thurwachter, Austria National Coordinating Centre, Austria
Ursula Wieder, Brustzentrum Hanusch-KH, Department Chirurgie/Gynäkologie , Brustzentrum Hanusch-KH, Department Chirurgie/Gynäkologie, Austria
Ehtesham Abdi, The Tweed Hospital, New South Wales, Australia
Sandra Allen, Nambour, Queensland, Australia
Test Anzmco, Newcastle, Australia
Anne Arruzza, Liverpool, New South Wales, Australia
Heath Badger, Dept of Surgical Oncology, ANZBCTG, Newcastle, Australia
Caroline Baker, Mercy Private Hospital / Victorian Breast & Oncology Care, Victoria, Australia
Geoffrey Beadle, Royal Brisbane Hospital, Queensland, Australia
Ian Bennett, Princess Alexandra Hospital, Queensland, Australia
Angela Benson, Victorian Breast & Oncology Care / Mercy Private Hospital, Victoria, Australia
Robert Blum, Bendigo Health, Victoria, Australia
Adam Boyce, Lismore Base Hospital, New South Wales, Australia
Fran Boyle, Mater North Sydney, North Sydney, Australia
Karen Briscoe, Coffs Harbour Health Campus, New South Wales, Australia
Kelsey Bumford, Peter MacCallum Cancer Centre, Victoria, Australia
Sonia Byrne, Lismore Base, New South Wales, Australia
Jenny Campbell, Royal Brisbane, Queensland, Australia
Hugh Carmalt, New South Wales, Australia
Margaret Chamen, Tamworth Rural Referral Hospital, New South Wales, Australia
Anupam Chaudhuri, Riverina Cancer Care Centre - DCIS, New South Wales, Australia
Sharon Clark, The Tweed Hospital, New South Wales, Australia
David Clark, The Breast Centre, New South Wales, Australia
Amy Clark, Bendigo Health, Victoria, Australia
Vicki Clowes, Goulburn Valley, Victoria, Australia
John Collins, Royal Melbourne Hospital, Melbourne, Australia
Annette Cubitt, Royal Brisbane, Queensland, Australia
Giuliana D'Aulerio, Sir Charles Gairdner, Western Australia, Australia
Rachel Dear, St Vincents Sydney, New South Wales, Australia

Stephen Della-Fiorentina, Southern Highlands, New South Wales, Australia
Stephen Della-Fiorentina, Southern Highlands, New South Wales, Australia
Jennifer Donovan, Royal North Shore Hospital, New South Wales, Australia
Michael Donovan, Nambour General Hospital, Queensland, Australia
Karin Dunne, The Tweed, New South Wales, Australia
Jane Eade, Griverina cancer care centre, New South Wales, Australia
Emma Eagles, Southern Highlands Cancer Centre, New South Wales, Australia
Melissa Fox, Macarthur, New South Wales, Australia
Nicole Francis, Breast Cancer Trials, Newcastle, Australia
Sonya Gibbons, Lismore Base, New South Wales, Australia
Peter Grantley Gill, Royal Adelaide Hospital, South Australia, Australia
Rebecca Griffiths, Tamworth Rural Referral Hospital, New South Wales, Australia
Donna Haberl, Victoria, Australia
Stewart Hart, Monash Medical Centre, Victoria, Australia
Terri Hartweger, Monash University, Eastern Clinical School, Victoria, Australia
Danielle Harward, Southern Highlands, New South Wales, Australia
Claire Haworth, Sr Charles Gairdner, Western Australia, Australia
Jasmine Hee, St Vincents, Melbourne, Victoria, Australia
Jane Hill, Riverina Cancer Care Centre, New South Wales, Australia
Rosemary Hurley, ANZNB, New South Wales, Australia
Jeralyn Jacquet, Southern Highlands, New South Wales, Australia
Jennifer Jagoe, Southern Highlands, New South Wales, Australia
Catherine Johnso12, Austin Health, Victoria, Australia
Lynne Jolly, St Vincent's Sydney, New South Wales, Australia
David Joseph, Sir Charles Gardiner Hospital, Western Australia, Australia
George Kannourakis, Victoria, Australia
Sophie Katsabanis, Peter MacCallum, Victoria, Australia
Lauren Keller, Melbourne, Australia
Charlene Kobas, Princess Alexandra, Queensland, Australia
Carolyn Kwong, Royal North Shore, New South Wales, Australia
Mari Lashbrook, Riverina, New South Wales, Australia
Michael Law, Maroondah, Victoria, Australia
Ingrid Laycock, Breast Cancer Trials, Newcastle, Australia
Lisa Leopardi, Royal Adelaid, South Australia, Australia
Marian Lieschke, Royal Melbourne, Melbourne, Australia
Jennifer Liu, Victoria, Australia
Yali Liu, Austin Health, Victoria, Australia
Mona Martyn-Smith, Mater, North Sydney, Australia
Gavin Marx, San Clinical Trials, New South Wales, Australia
Richard Masters, Box Hill Hospital, Victoria, Australia
Andrea Mckenzie, Royal Brisbane, Queensland, Australia
Narelle Mcphee, Bendigo Health, Victoria, Australia
Lauren Mitchell, Victoria, Australia
Carole Mott, Goulburn Valley, Victoria, Australia
Eugene Moyland, Liverpool, New South Wales, Australia
Michelle Nottage, Royal Brisbane, Queensland, Australia
Felicity Osmond, Victoria, Australia
Nick Pavlakis, Armidale Hospital, New South Wales, Australia
Kelly-Anne Phillips, Peter Mac Callum Cancer Centre, Victoria, Australia
Annabel Pickett, Coffs Harbour, New South Wales, Australia
Samara Price, Macarthur Cancer Therapy Centre, New South Wales, Australia
Wendy Pritchard, Royal Brisbane, Queensland, Australia
Nadia Ranieri, St Vincent's Melbourne, Victoria, Australia
Kathryn Rebellato, New South Wales, Australia
Jessica Reid, Royal Adelaide Hospital, South Australia, Australia
Hollie Ritchie, Calvary Mater Newcastle, New South Wales, Australia
Natasha Roberts, Royal Brisbane, Queensland, Australia
Kayte Robinson, Victorian Breast & Oncology Care/Mercy Private Hospital, Victoria, Australia
Rachael Rowse, The Tweed Hospital, New South Wales, Australia
Amanda Rundle, Bendigo Health, Victoria, Australia

Suzanne Ryan, Nambour, Queensland, Australia
Suma Santhosh, Macarthur, New South Wales, Australia
Frank Sardelic, Tamworth Hospital, New South Wales, Australia
Christobel Saunders, Sir Charles Gairdner Hospital, Western Australia, Australia
Eva Segelov, New South Wales, Australia
Kimberley Sheather, Mater, North Sydney, Australia
Judith Silcock, Breast & Endocrine Centre, New South Wales, Australia
Cassandra Simon, Coffs Harbour Health Campus; ANZNC, New South Wales, Australia
Nina Singh, San Clinical Trials, New South Wales, Australia
Joanne Smith, Coffs Harbour, New South Wales, Australia
Raymond Snyder, St Vincent's Hospital Melbourne, Victoria, Australia
Victoria Sproule, Calvary Mater Newcastle, New South Wales, Australia
Amy Tang, Box Hill Hospital, Victoria, Australia
Javier Torres, Goulburn Valley, Victoria, Australia
Krystine Walsh, Maroondah, Victoria, Australia
Shane White, Austin Heath, Victoria, Australia
Stephen Wilkinson, Royal Hobart Hospital, Hobart, Australia
Nicholas Zdenkowski, New South Wales, Australia
Inneke Bambust, Dendermonde, Belgium
Barbara Beier, Brussels, Belgium
Martine Berliere, Brussels, Belgium
Nathalie Blondeel, Brussels, Belgium
Magda Boels, Borstkliniek,Laarbeeklaan, Brussel, Belgium
Ilse Claessens, Aalst, Belgium
Dominique Crasson, Namen, Belgium
Lionel D'Hondt, Godinne, Belgium
Luc Dirix, Wilrijk, Belgium
Frederic Forget, Libramont, Belgium
Francoise Henry, Brussels, Belgium
Marc L'Hermite, Brussels, Belgium
Jan Lamote, Borstkliniek,Laarbeeklaan, Brussel, Belgium
Fabienne Lieben, Brussels, Belgium
Patrick Neven, University Hospitals, Leuven, Belgium
Jean-Marie Nogaret, Rue Héger Bordet 1 Brussels, Brussels, Belgium
Guy Orye, Campus Virga Jesse, Hasselt, Belgium
Heidi Roelstrate, Aalst, Belgium
Monique Seret, Namen, Belgium
Phillipe Simon, Brussels, Belgium
Daisy Supply, University Hospitals Leuven, Leuven, Belgium
Marijke Tollenaers, Secretariaat gynaecologie, Genk, Belgium
Sofie Tombeur, Aalst, Belgium
Ann Tullen, Hasselt, Belgium
Jos Vlasselaer, Genk, Belgium
Sophie Vrancken, Wilrijk, Belgium
Margit Bachmann, Thun, Switzerland
Barbara Bolliger, Tumor- und Brustzentrum ZeTuP, Switzerland
Markus Borner, Bern, Switzerland
Farzaneh Borner, Inselspital Bern Universitatsklinik fur Medizinische Onkologie, Bern, Switzerland
Susanne Bucher, Luzerner Kantonsspital, Neue Frauenklinik Brustzentrum, Switzerland
Pierre O. Chappuis, Unité d'oncogénétique et de prévention des cancers, Service d'Oncologie, HUG , Genève, Switzerland
Bénédicte Fleury, Centre Pluridisciplinaire d'Oncologie, CHUV - PAV 02-12, Genève, Switzerland
Marie Galmiche, Switzerland
Agnes Glaus, Centre for Tumour Detection and Prevention, Switzerland
Claudia Guebelin, Bern, Switzerland
Stephanie Largiader, Switzerland
Olivia Pagani, IOSI - Ospedale Regionale Bellinzona e Valli, Switzerland
Manuela Rabaglio, Klinik und Poliklinik für Medizinische Onkologie, Inselspital Bern, Bern, Switzerland
Daniel Rauch, Onkologiezentrum Thun, Spital STS AG, Thun, Switzerland
Khalil Zaman, Centre pluridisciplinaire d‘oncologie, CHUV, Switzerland

Marcela Fritis, Fundacion Arturo Lopez Perez, Santiago, Chile, Chile
Jorge Gamboa, Santiago, Chile, Chile
Bettina Muller, Chilean Cooperative Group for Oncologic Research (GOCCHI), Chile National Coordinating Centre, Chile
Octavio Peralta, c/o GOCCHI, Chile National Coordinating Centre, Chile
Ricardo Schwartz, Hospital Militar, Santiago, Chile, Chile
Sebastian Sole, Santiago, Chile, Chile
Zdenka Zlatar, Chilean Cooperative Group for Oncologic Research (GOCCHI), Chile National Coordinating Centre, Chile
Oberhoff, Katholische Kliniken Essen-Nord, Germany
B. Aktas, Germany
Andrea Stefek, Germany
Anke J. Kleine-Tebbe, Germany
Doris Augustin, Klinikum Deggendorf, Mammazentrum, Germany
Baake, Onkologische Gemeinschaftspraxis, Germany
Baerens, Frauenarztpraxis, Frauenheilkunde und Geburtshilfe, Germany
Leila Bauer, Germany
Baumann, Universitätsklinikum, Frauenklinik Mutter-Kind-Zentrum, Germany
Beldermann, Germany
Michael Berghorn, Germany
Peter/ Bern Schleicher, Praxis Dr. Bern + Peter Schleicher, Germany
Breitbach, Krankenhaus Neunkirchen GmbH, Germany
Brucker, Klinikum Nuernberg, Brustzentrum, Germany
Oa Camara, Universitätsklinikum Jena, Germany
Klaus Christl, Krankenhaus Eggenfelden, Frauenklinik, Germany
Christoph Mundhenke, Universitaetsklinikum Schleswig-Holstein, Campus Kiel, Germany
Dall, Germany
Mustafa Deryal, Germany
Doreen Düchting, Germany
Elena Langer, Germany
G Emons, Universitätsklinikum, Göttingen, Germany
Eva Stauß, Germany
Petra Feer, GBG Forschungs GMBH, German National Coordinating Centre, Germany
Gerhard Nohe, Germany
Ioannis Gkantiragas, GBG Forschungs GmbH, German National Coordinating Centre, Germany
Karsten Gnauert, Aalen, Germany
Kay Goerke, Germany
Sabine Gross, Marienhospital, Stuttgart, Germany
Göhring, Johanniter-Krankenhaus, Frauenklinik, Germany
Kornelia Göhring, Germany
Hans-Christian Kolberg, Marienhospital, Gynaekologie und Geburtshilfe, Germany
Heinrich, Praxis Dr. Heinrich, Onkol. Schwerpunktpraxis fuer Gynaekologie, Germany
M. Herwig, Germany
Ursula Hille, Medizinische Hochschule, Brustzentrum, Germany
Hindenburg, Gynäkologisch-Onkologische Schwerpunktpraxis, Germany
Thomas Hitschold, Germany
Hoeffkes, Klinikum Fulda, MVZ Osthessen/Studienbuero, Germany
Hoffmann, St. Josefshospital, Germany
Kaufmann, Universitaetsklinikum, Frauenklinik, Germany
P Klare, Praxisklinik Krebsheilkunde für Frauen, Germany
Philip Knapp, German National Coordinating Centre, Germany
Koehne, Klinikum Oldenburg, Klinik fuer Innere Medizin II/Onkol. Tagesklinik, Germany
Petra Krabisch, Klinikum Chemnitz, Germany
Uwe Kullmer, Germany
G Köhler, Universitätsklinikum, Greifswald, Germany
C.-H. Köhne, Germany
H Kölbl, Universitätsklinikum Mainz, Germany
Sabine Lemster, Germany
L. Liedtke, Germany
Cornelia Liedtke, Germany

Lindner (Coop Carstensen), Krankenhaus Elim, Germany
Frederik Marmé, Universitatsklinikum Heidelberg, NCT, Germany
B. Ataseven, Germany
Gunter Von Minckwitz, GBG Forschungs GMBH, German National Coordinating Centre, Germany
Splitt, Facharzt f. Frauenheilkunde u. Geburtshilfe, Germany
Meinerz, St. Vincenz Krankenhaus GmbH, Gynaekologische Ambulanz, Germany
Volkmar Müller, Germany
Thomas Noesselt, Kreiskrankenhaus Hameln, Frauenklinik/Onkol. Ambulanz, Germany
Paepke, Klinikum rechts der Isar der Techn. Univ. München, Germany
Pourfard/Uleer, Gemeinschaftspraxis Hildesheim, Frauenheilkunde und Geburtshilfe, Germany
Bauer, Brustzentrum Suedbaden, Germany
Beckmann, Friedrich-Alexander-Universität, Germany
Costa, Otto-von-Guericke-Universitaet, Germany
Distler, Technische Universität Dresden, Germany
Sommer, I. Universitäts-Frauenklinik, Germany
Thomssen, Klinikum der Med. Fakultät, Germany
Tulusan, Klinikum Bayreuth, Germany
Ulmer, Staedt. Klinikum Karlsruhe, Germany
Andreas Rempen, Schwäbisch Hall, Germany
Rezai, Brustzentrum Luisenkrankenhaus Duesseldorf, Germany
Rita Schmutzler, Universitaetsklinikum Koeln, Frauenklinik, Germany
Henning Ritter, Pius-Hospital Oldenburg, Frauenklinik, Germany
Scharl, Klinikum St. Marien Amberg, Germany
Schindelin, Dr.-Horst-Schmidt-Kliniken GmbH, Germany
Sabine Schmatloch, Germany
A Schneider, Germany
Claudia Schumacher, St. Elisabeth Krankenhaus, Brustzentrum Koeln-Hohenlind, Germany
Schwenzer, Klinikum Dortmund, Frauenklinik, Germany
Anne Schwiebus, Germany
Stephan Seitz, Germany
Thomas Steck, Klinikum Passau, Frauenklinik, Germany
Dirk Strumberg, Germany
Tesch, Onkologie Bethanien/Marien-Krankenhaus, Germany
Toralf Reimer, Germany
Volker Hanf, Klinikum Fuerth, Frauenklinik, Germany
Erich Weiss, Klinikum Sindelfingen-Boeblingen GmbH, Brustzentrum der Frauenklinik, Germany
W Weist, Katholisches Klinikum Mainz/St. Vinzenz, Germany
Wolfgarten, Germany
Zahm, Brustzentrum Ostthueringen am SRH Waldklinikum Gera, Germany
Louise Bjørn, AALBORG SYGEHUS, Denmark
Charlotte Lanng, Herlev University Hospital, Denmark
Jens Peter Garne, Aalborg Hospital, Breast Surgery Department, Denmark
Tiina Palva, Pirkanmaa Cancer Society, Tampere, Finland
Marjo Virkki, Tampere/Finland, Tampere, Finland
Leslie Ardilouze, Institut Bergonié, Bordeaux, France
Aude Barbier, Institut Gustave Roussy, Villejuif, France
Célia Baylac, France
Ahmed Benyoucef, Centre Henri Becquerel, Rouen, France
Jean Pierre Bergerat, Département Hémato-Oncologie, France
Olivier Bernard, Agen, France
Dominique Berton-Rigaud, ICO René Gauducheau, Nantes, France
Virginie Birr, Hôpital Emile Muller, France
Gabrielle Blanche, CENTRE PAUL STRAUSS, France
Nathalie Bonichon - Lamicchane, CT Bordeaux, France
Delphine Brunie, France
Abdesslam Chajara, Brest, France
Céleste David, Institut Sainte Catherine, France
Francesco Del Piano, France
Sengul Deveci, France
Nadine Dohollou, France

Claire Dovergne, Unicancer, Paris FNCLCC, France
Anne-Marie Elgard-Maitre, Hôpital Emile Muller, France
Myriam Estrabaut, Institut Claudius Regaud, France
Hélène Etienne, Hôpitaux Universitaires de Strasbourg, France
Jean-Christophe Eymard, Institut Jean Godinot, Reims, France
Marie-Josèphe Foucher-Goudier, Centre Hospitalier de Bretagne Sud, France
Danièle Fric, Daniel Institute Hollard Level 1, France
Sara Garcia, Paris FNCLCC, France
Sylvia Giard-Lefevre, Centre Oscar Lambret, Lille, France
Laurence Gladieff, Institut Claudius Regaud, France
Anais Grilo, Centre Georges-François Leclerc, Dijon, France
Hayat Guezi, Brest, France
Flora Hernandez, France
Mahmoud Ibrahim, CENTRE HOSPITALIER RÉGIONAL D'ORLÉANS, France
Gaelle Kergoat, Centre Eugène Marquis, Rennes, France
Gaëlle Landry, France
Mariella Le Saux, Centre Hospitalier de Bretagne Sud, France
Bérengère Legendre, Centre Oscar Lambret, Lille, France
Christelle Levy, Center François Baclesse, Caen, France
Hélène Maduzio, Unicancer, Paris FNCLCC, France
Hugo Marsiglia, Institut Gustave Roussy, Villejuif, France
Adina Marti, France
Severine Maury, ICO René Gauducheau, Nantes, France
Reda Mesnaria, Centre Hospitalier de Lagny, Lagny-sur-Marne, France
Brahim Ouahrani, CENTRE HOSPITALIER RÉGIONAL D'ORLÉANS, France
Corinne Penaud, R&D UNICANCER, France
Lynda Pervieux, Hôpital Dupuytren, France
Erouan Petit, Centre Georges-François Leclerc, Dijon, France
Thierry Petit, Centre Paul Strauss, France
Giles Piot, Clinique des Ormeaux, France
Cassandra Porebski, France
Lucie Randazzo, Agen, France
Olivier Rastelli, Centre Henri Becquerel, Rouen, France
Armelle Rollet, Institut Sainte Catherine, France
Daniel Serin, Institut Sainte-Catherine, France
Héléne Simon Swirsky, Brest, France
Valery Tardy, Clinique des Ormeaux, France
Caroline Toussaint, Centre Hospitalier de Lagny, Lagny-sur-Marne, France
Magali Tsachiris, CT Bordeaux, France
Nicole Tubiana-Mathieu, Hôpital Dupuytren, France
Christine Tunon De Lara, Institut Bergonié, Bordeaux, France
Aude Willerval, France
Abigail Alford, Breast Cancer Unit, Castle Hill Hospital, Cottingham, United Kingdom
Amit Bahl, Bristol RI, United Kingdom
Rehana Bakawala, Cheltenham General Hospital, Cheltenham, United Kingdom
Jenny Baker, Cancer Research Team, York, United Kingdom
Rachel Barnsley, Research & Innovation, Poole, United Kingdom
Urmila Barthakur, Clinical Oncology, Yeovil, United Kingdom
Imogen Batty, Surgical Directorate, Royal Bournemouth Hospital, Bournemouth, United Kingdom
Kate Beesley, Clinical Research Unit, Yeovil, United Kingdom
Gian Filippo Bertelli, Singleton Clinical Trials Unit, Singleton Hospital, Swansea, United Kingdom
Sarah Bevins, Bristol Breast Care Centre, Bristol, United Kingdom
Kirsty Bracewell, Brighton, United Kingdom
Lynne Bradshaw, Bristol Breast Care Centre, Bristol, United Kingdom
Maria Bramley, Department of General Surgery, Oldham, United Kingdom
Lynne Breeze-Jones, South West Wales Cancer Institute, Swansea, United Kingdom
James Bristol, Department of Surgery, Cheltenham General Hospital, Cheltenham, United Kingdom
Hayley Brooks, Oncology Research Team, Manchester, United Kingdom
Nigel Bundred, South Manchester University Hospital, Academic Surgery, Manchester, United Kingdom
Lucy Cadmore, Plymouth, United Kingdom

Amitabha Chakrabarti, Dorchester, United Kingdom
Sankaran Chandrasekharan, Essex Rivers Healthcare Trust, Essex County Hospital, Colchester, United Kingdom
Jill Chittock, Gloucestershire Research Team, Focus Research Centre, Cheltenham General Hospital, Cheltenham, United Kingdom
Angela Chrisopoulou, The Nightingale Centre, Manchester, United Kingdom
Shirley Cocks, Clinical Research Team Davenport House, Bolton, United Kingdom
Karen Combe, Edinburgh Breast Unit, Edinburgh, United Kingdom
Hilary Congdon, Oncology Clinical Trials Unit, Plymouth, United Kingdom
Adam Critchley, Newcastle-Upon-Tyne, United Kingdom
Andrea Croucher, Frimley, United Kingdom
Helen Cumming, Stefani Unit, Ninewells Hospital, Dundee, United Kingdom
Ramsey Cutress, Cancer Research UK Centre, Southampton, United Kingdom
Mariella D'Alessandro, Clinical Genetics Centre, Aberdeen, United Kingdom
Raouf Daoud, Frimley Park Hospital NHS Trust, Frimley, United Kingdom
Eileen Dillon, Belfast, United Kingdom
Tracey Dobson, Oncology Research - Queen Alexandra Hospital, Portsmouth, United Kingdom
Susan Downer, Royal Devon and Exeter Hospital, Exeter, United Kingdom
Philip Drew, Royal Cornwall Hospital, Truro, United Kingdom
Sidharth Dubey, Derriford Hospital, Plymouth, United Kingdom
Jackie Elliott, Queens Hospital Burton, Burton-on-Trent, United Kingdom
Gareth Evans, Regional Genetic Service, St Mary's Hospital, Manchester Prev, United Kingdom
Abigail Evans, Poole, United Kingdom
Kay Facey, Clinical Trials Unit, Plymouth Oncology Centre, Plymouth, United Kingdom
Douglas Ferguson, Room H266, Royal Devon and Exeter Hospital, Exeter, United Kingdom
Mary Fitzpatrick-Greening, Oncology & Haematology Clinical Trials (OHCT), Guys London, United Kingdom
Jo Fletcher, Lincolnshire Clinical Research Facility, Lincoln Pilgrim, United Kingdom
Olesya Francis, Lincolnshire Clinical Research Facility, Research & Innovation, Lincoln, United Kingdom
Sarah Funnell, Cancer Trials Office, Worthing, United Kingdom
Fiona Geddes, Breast Unit - Western General Hospital, Crewe South Road, Edinburgh, United Kingdom
Sian Gibson, Oncology Research Department, Chelmsford, United Kingdom
Kayleigh Gilbert, Whittington, United Kingdom
Jeanette Gilbert, Worthing, United Kingdom
Jemma Gilmore, Oncology Research Team, Welwyn Garden, United Kingdom
Lynn Glass, Cancer Clinical Trials, Wishaw, United Kingdom
Sarah Goodwin, Sussex Cancer Research Team, East Sussex, United Kingdom
Anne Griffiths, Truro, United Kingdom
Lwazi Grinly, Clinical Trials Unit, Plymouth Oncology Centre, Plymouth, United Kingdom
Nabina Gurung, Oncology Research, Office Research & Innovation Department, Poole, United Kingdom
Eleanor Gutteridge, NCCTT Group 1, Nottingham, United Kingdom
Hisham Hamed, Academic Oncology Unit, Bermondsey Block, Guys London, United Kingdom
Claudia Harding-Mckean, Ursula Keyes Breast Unit, Countess of Chester Hospital, Chester, United Kingdom
Annamaria Harmathova, Clinical Research Coordinator I Research & Development, Northwick Park, United Kingdom
Susan Hartup, Breast Care Research Nurses, St James's University Hospital, Leeds, United Kingdom
Sarah Hathaway-Lees, Queens Hospital Burton, Burton-on-Trent, United Kingdom
Amy Henson, Breast Care Research Nurses, St James's University Hospital, Leeds, United Kingdom
Verity Henson, Clinical Trials Unit, Bristol RI, United Kingdom
Naomi Hill, Belfast, United Kingdom
Annette Hilldrith, United Lincolnshire Hospitals NHS Trust - Greetwell Road, Lincoln, United Kingdom
Zoe Hilton, Breast services, Cardiff, United Kingdom
Katy Hoare, Lincolnshire Clinical Research Facility Research and Innovation, Lincoln, United Kingdom
Karen Hogben, Dorchester, United Kingdom
Chris Holcombe, Breast Unit, Linda McCartney Centre, Royal Liverpool University Hospital, Liverpool, United Kingdom
Kieran Horgan, St James's University Hospital, Leeds, United Kingdom
Helen Hothersall, Ward 12 Research Office, Airedale NHS Foundation Trust, Keighley, United Kingdom
Tony Howell, Manchester, United Kingdom
Naila Ihsan, Cottingham, United Kingdom
Hayley Inman, Bradford Institute of Health Research, Bradford, United Kingdom

Shabana Iqbal, Huddersfield, United Kingdom
Sejal Jain, Division 1 - Cancer, London, United Kingdom
Karen Janes, Linda McCartney Centre, Royal Liverpool University Hospital, Liverpool, United Kingdom
Jibril A Jibril, Lincoln County Hospital -Greetwell Road, Lincoln, United Kingdom
Amy Kirkby, Lincolnshire Clinical Research Facility, Lincoln Pilgrim, United Kingdom
Peter Kneeshaw, Castle Hill Hospital, Cottingham, United Kingdom
Jalal Kokan, Macclesfield, United Kingdom
Justyna Kukielska, Gloucestershire Research Team, Cheltenham, United Kingdom
Alison Lannigan, Wishaw General Hospital, Wishaw, United Kingdom
Mark Lansdown, St James's University Hospital, Leeds St.James, United Kingdom
Tara Lawrence, Lincoln Pilgrim, United Kingdom
Yvonne Lester, Chelmsford, United Kingdom
Carla Lewis, Worthing, United Kingdom
Rick Linforth, Breast Unit, Bradford, United Kingdom
Nicola Lunt, Macclesfield District General Hospital, Macclesfield, United Kingdom
Simon Marsh, Breast Unit, Colchester, United Kingdom
Donna Mcevoy, Newcastle-Upon-Tyne, United Kingdom
Stuart Mcintosh, Belfast, United Kingdom
Deborah Melia, Oncology Research Department, Huddersfield Royal Infirmary, Huddersfield, United Kingdom
Clare Mewies, Burton Hospitals NHS Foundation Trust, Burton-on-Trent, United Kingdom
Zosia Miedzybrodzka, Clinical Genetics Centre, Aberdeen, United Kingdom
Sara Millen, Dundee, United Kingdom
Adrian Moss, Breast Clinic, Worthing, United Kingdom
Helen Munday, Southwick Hill Rd, Cosham, Portsmouth, United Kingdom
Claire Murphy, Airedale General Hospital, Keighley, United Kingdom
Eisa Nael, Stafford, United Kingdom
Shirin Namini, Clinical Research Team, Wakefield, United Kingdom
Vitalis Nwokorie, CRN: South London (Oncology), London, United Kingdom
Lynn Osborne, United Lincolnshire Hospitals NHS Trust, Grantham, United Kingdom
Julie Pascoe, Oncology Clinical Trials Unit, Plymouth, United Kingdom
Ashraf Patel, Breast Unit, Epping, United Kingdom
Jess Perry, Yeovil, United Kingdom
Mojca Persic, Burton-on-Trent, United Kingdom
Andrew Proctor, Research & Development Unit, York, United Kingdom
Sabina Rachid, Northwick Park, United Kingdom
Rashika Rajakumar, Whittington, United Kingdom
Ridha Ramiz, Research Team, Huddersfield, United Kingdom
Kerry Rennie, The Clinical Trials Unit, Yeovil District Hospital, Yeovil, United Kingdom
Nathalie Rich, Cancer Research Delivery Group, London, United Kingdom
Lucia Richardson, Academic Oncology Department, Cottingham, United Kingdom
Helen Robertshaw, Bradford Institute for Health Research, Bradford, United Kingdom
Nicola Roche, Royal Marsden Hospital, London, United Kingdom
Michelle Saull, Whittington, United Kingdom
Sarah Scourfield, Cardiff, United Kingdom
Elizabeth Shah, East Sussex Hospitals NHS Trust, The Ridge, East Sussex, United Kingdom
Mike Shere, Breast Care Centre, Southmead Hospital, Bristol, United Kingdom
Mark Sibbering, Department of General Surgery, Royal Derby Hospital, Derby, United Kingdom
Anthony Skene, Royal Bournemouth Hospital, Bournemouth, United Kingdom
Simon Smith, Breast Unit, Chelmsford, United Kingdom
Jill Stacey, Staffordshire General Hospital, Stafford, United Kingdom
Kim Stevens, Duthie building MP306, Southampton, United Kingdom
Helen Sweetland, The Breast Centre, Cardiff, United Kingdom
Glyn T Neades, Breast Unit - Western General Hospital, Crewe Road South, Edinburgh, United Kingdom
Jo-Anne Taylor, Conquest Hospital, The Ridge, East Sussex, United Kingdom
Issy Thomas, Lincolnshire Clinical Trials Unit, Pilgrim Hospital, Lincoln Pilgrim, United Kingdom
Alastair Thompson, Department of Surgical Oncology, Division of Surgery, Dundee, United Kingdom
Jonathan Thompson, NI Cancer Clinical Trials Unit East Podium, 'C' Floor, Belfast City Hospital, Belfast, United Kingdom
Sarah Thorpe, The Oncology Research Team, The Nightingale Centre, Manchester, United Kingdom
Jayant Vaidya, The Clinical Trials Group, Royal Free and UCL Medical School, Whittington, United Kingdom

Philip Walker, Bolton, United Kingdom
Sarah White, SWLCRN, London, United Kingdom
Caroline Wilson, Sheffield, United Kingdom
Virginia Wolstenholme, Department of Clinical Oncology, London, United Kingdom
Jeremy Wood, QE2 Breast Unit, Welwyn Garden, United Kingdom
Charles Zammit, Nigel Porter Unit, Royal Sussex County Hospital, Brighton, United Kingdom
Zsuzsanna Kahan, Department of Oncotherapy, University of Szeged, Szeged, Hungary
Brigitta Éberling, University of Szeged, Department of Onkotherapy, Szeged, Hungary
Ashley Bazin, Dept. Of Medical Oncology, Tallaght, Ireland
Margaret Burke, Oncology/Haematology Clinical Trials, Sligo, Ireland
Tara Byrne, Cancer Trials Ireland, Dublin, Ireland
Mary Doyle, UC Dublin, Ireland
Denis Evoy, ICORG, the Irish Clinical Oncology Research Group, UC Dublin, Ireland
Sharon Gardiner, Limerick, Ireland
Rajnish Gupta, ICORG, the Irish Clinical Oncology Research Group, Limerick, Ireland
Aisling Hegarty, Department of Surgery, Beaumont Hospital Beaumont, Beaumont Dublin, Ireland
Arnold Hill, ICORG, the Irish Clinical Oncology Research Group, Dublin, Ireland
Marian Jennings, Oncology Clinical Trials Office, University Hospital Galway, Galway, Ireland
John Kennedy, ICORG, the Irish Clinical Oncology Research Group, Dublin, Ireland
Michael Kerin, ICORG, the Irish Clinical Oncology Research Group, Galway, Ireland
Ingrid Kiernan, Oncology Department, Dublin, Ireland
Michael J. Martin, ICORG, the Irish Clinical Oncology Research Group, Sligo, Ireland
Elaine Mccarthy, Limerick, Ireland
Deirdre O'Hanlon, ICORG, the Irish Clinical Oncology Research Group, Cork, Ireland
Debra O'Hare, Cork, Ireland
Kathleen Scott, Dublin, Ireland
Janice Walshe, ICORG, the Irish Clinical Oncology Research Group, Tallaght, Ireland
Gian Antonio Da Prada, Medical Oncology Division, Italy
Fabrizio Artioli, Ospedale B. Ramazzini, V. Guido Molinari, Italy
Antonio Bernardo, Istituti clinici scientifici Maugeri, Italy
Andrea Bianchetti, Italy
Bernardo Bonanni, Division of Chemoprevention, Italy
Fulvio Borella, Italy
Elisabetta Cretella, Italy
Giuseppe De Nittis, Italy
Massimo Federico, Azienda Ospedaliera Universitaria die Modena, Modena, Italy
Nicola Flego, Florence DCIS, Italy
Daniele Generali, Cremona ITALY, Italy
Lorenzo Gianni, Divisione Oncologia Ed Onco-Ematologa, Italy
Claudio Graiff, Azienda Sanitaria di Bolzano, Italy
Aliana Guerrieri Gonzaga, Division of Chemoprevention, Italy National Coordinating Centre, Italy
Annalisa Lanza, Italy
Maria Michiara, Azienda Ospedaliero-Universitaria di Parma, Italy
Mara Negri, Servizio Data Management, Italy National Coordinating Centre, Italy
Lorenzo Orzalesi, University of Florence, Florence, Italy
Elisa Picardo, Italy
Graziella Pinotti, Italy
Riccardo Ponzone, Italy
Mauro Porpiglia, Aso O.I.R.M. Sant'anna, Italy
Elena Rapacchi, Italy
Giorgia Razzini, Italy
Jenny Roselli, Florence, Italy
Britt Rudnas, Italy
Claudia Sangalli, Servizio Data Management, Italy National Coordinating Centre, Italy
Edda Simoncini, Italy
Carlo Tondini, Ospedali Riuniti Di Bergamo, Italy
Ilaria Vallini, Italy
Clara Varricchio, Italy National Coordinating Centre, Italy
Barbara Venturini, Italy
Marilena Visini, Azienda Ospedaliera Ospedale di Lecco, Italy

Stephen Brincat, Oncology Day Unit, Tal Qroqq, Malta, Malta
Nadia Cilia, Oncology Day Unit, Tal Qroqq, Malta, Malta
Ian Campbell, Waikato Hospital, New Zealand, Hamilton, New Zealand
Heather Flay, Waikato Hospital, Hamilton, New Zealand
Brendan Luey, Wellington, Wellington, New Zealand
Michael Mccrystal, North Shore Hospital Auckland, New Zealand , Auckland, New Zealand
Debra Morriss, Wellington, Wellington, New Zealand
Kathryn Neilson, St George's, Christchurch, New Zealand
Sherry Nisbet, North Shore, Auckland, New Zealand
Gill Rolfe, Auckland, New Zealand
Jenni Scarlet, Waikato, Hamilton, New Zealand
Andrew Simpson, Wellington Hospital, Wellington, New Zealand
Nic Stevens, St Georges, Christchurch, New Zealand
Chris Wynne, Canterbury Breast Care (Christchurch), Christchurch, New Zealand
Conceicao Costa, Gabinete de Investigação Clínica, Lisbon, Portugal
Paula Rodrigues, Gabinete de Investigação Clínica, Lisbon, Portugal
Gabriela Sousa, Lisbon, Portugal
Fatima Vaz, Instituto Portugues De Oncologia, Gabinete De Estudos Clinicos, Lisbon, Portugal
Linda Agren, Lund, Sweden
Anna-Karin Falck, Department of Surgery, Helsingborg, Sweden
Kristina Johansson, Swedish Monitors, Sweden
Jenny Johansson, Stockholm, Sweden
Jakob Kaij, Department of Surgery, Skånes Universitetssjukhus Department of Surgery, Sweden
Sara Margolin, Department of Oncology, Stockholm, Sweden
Kerstin Reistad, Helsingborg, Sweden
Anita Ringberg, Malmo University Hospital, Swedish Monitors, Sweden
Lisa Rydén, Department of Surgery, Lund, Sweden
Lisa Rydén, Department of Surgery, Swedish Monitors, Sweden
Gunilla Sellerstam, Stockholm, Sweden
Jan Sundberg, Department of Oncology, Swedish Monitors, Sweden
Jan Sundberg, Department of Oncology, Lund, Sweden
Marie Sundqvist, Swedish Monitors, Sweden

IBIS-II coordinating centre:
Jessica Adams
Jane Hickman
Victoria Davis
Nadia Haidar
